# Minimal Out-Toeing and Good Hip Scores of Severe SCFE Patients Treated With Modified Dunn Procedure and Contralateral Prophylactic Pinning at Minimal 5-year Follow up

**DOI:** 10.1097/BPO.0000000000002127

**Published:** 2022-03-07

**Authors:** Till D. Lerch, Adam Boschung, Christiane Leibold, Roger Kalla, Hassen Kerkeni, Heiner Baur, Patric Eichelberger, Simon D. Steppacher, Emanuel F. Liechti, Klaus A. Siebenrock, Moritz Tannast, Kai Ziebarth

**Affiliations:** Departments of * Diagnostic, Interventional and Pediatric Radiology; † Orthopaedic Surgery; ‡ Neurology; ¶ Pediatric Surgery, Inselspital, Bern University Hospital, University of Bern; § Department of Physiotherapy, Bern University of Applied Sciences Health, Bern, Switzerland; ∥ Department of Orthopaedic Surgery and Traumatology, Fribourg Cantonal Hospital, HFR, University of Fribourg, Fribourg, Switzerland

**Keywords:** hip, slipped capital femoral epiphysis, gait analysis, hip joint, modified Dunn procedure, out-toeing

## Abstract

Slipped capital femoral epiphyses (SCFE) is associated with out-toeing of the foot and external rotation gait. But it is unknown if SCFE patients treated with the modified Dunn procedure have out-toeing at follow up.

Therefore, we used instrumented gait analysis and questioned (1) do severe SCFE patients treated with a modified Dunn procedure have symmetrical foot progression angle (FPA) compared with contralateral side and compared with asymptomatic volunteers (2) what is the prevalence of out-toeing gait and what are the outcome socres at follow up.

**Methods:**

Gait analysis of 22 patients (22 hips) treated with an unilateral modified Dunn procedure for severe SCFE (slip angle >60 degrees, 2002 to 2011) was retrospectively evaluated. Of 38 patients with minimal 5-year follow up, 2 hips (4%) had avascular necrosis of the femoral head and were excluded for gait analysis. Twenty-two patients were available for gait analysis at follow up (mean follow up of 9±2 y). Mean age at follow up was 22±3 years. Mean preoperative slip angle was 64±8 degrees (33% unstable slips) and decreased postoperatively (slip angle of 8±4 degrees). Gait analysis was performed with computer-based instrumented walkway system (GAITRite) to measure FPA with embedded pressure sensors. Patients were compared with control group of 18 healthy asymptomatic volunteers (36 feet, mean age 29±6 y).

**Results:**

(1) Mean FPA of SCFE patients (3.6±6.4 degrees) at follow up was not significantly different compared with their contralateral side (5.6±5.5 degrees) and compared with FPA of controls (4.0±4.5 degrees). (2) Of the 22 SCFE patients, most of them (19 hips, 86%) had normal FPA (−5 to 15 degrees), 2 patients had in-toeing (FPA<−5 degrees) and 1 had out-toeing (FPA >15 degrees) and was not significantly different compared with control group. (3) Mean modified Harris hip score (mHHS) was 93±11 points, mean Hip Disability and Osteoarthritis Outcome Score (HOOS) score was 91±10 points. Three patients (14%) had mHHS <80 points and walked with normal FPA. The 2 patients with in-toeing and one patient with out-toeing had mHHS >95 points.

**Conclusions:**

Patients with severe SCFE treated with modified Dunn procedure had mostly symmetrical FPA and good hip scores at long term follow up. This is in contrast to previous studies. Although 1 patient had out-toeing and 2 patients had in-toeing at follow up, they had good hip scores.

**Level of Evidence:**

Level III—retrospective comparative study.

Slipped capital femoral epiphysis (SCFE) is a common pediatric hip disease. An inferior and posterior displacement of the capital epiphysis is typical for SCFE in this very young patient group. A severe SCFE was defined by a slip angle of ≥60 degrees according to Southwick.^1^ SCFE is long-known hip disease associated with out-toeing gait (external rotation gait). SCFE can be treated either with in situ pinning or reorientation procedures such as the modified Dunn procedure. Residual deformities after in situ pinning especially in severe capital slips are can lead to femoroacetabular impingement^2^ and premature osteoarthritis.^3^,^4^ At long term follow up, the modified Dunn procedure for treatment of patients with severe SCFE can restore normal hip function, normal range of motion, and improved hip scores.^5^ But it is unknown if this procedure can restore normal foot progression angle (FPA) in SCFE patients.

Gait disorders including the in-toeing gait is a common cause for consultations for many pediatric orthopaedic surgeons. In-toeing of the foot is associated with increased femoral version, while out-toeing is associated with decreased femoral version.^6^ Furthermore, in-toeing gait could be a compensatory mechanism in patients with elevated femoral version to restore normal hip abductor force during walking.^7^ In-toeing can be present in children with Perthes’ disease,^8^ and in children with isolated elevated femoral version.^9^ Out-toeing gait can affect children with Perthes’ disease^8^ and with SCFE.^10^

But it is unknown if patients with severe SCFE have postoperative out-toeing of the foot or normal FPA after the modified Dunn procedure. Therefore, we used instrumented gait analysis and questioned:

1. Do severe SCFE patients treated with a modified Dunn procedure have symmetrical FPA compared with the contralateral side and compared with asymptomatic volunteers.
2. What is the prevalence of out-toeing gait.
3. What are the outcome scores at follow up.

## METHODS

**FIGURE 1:**
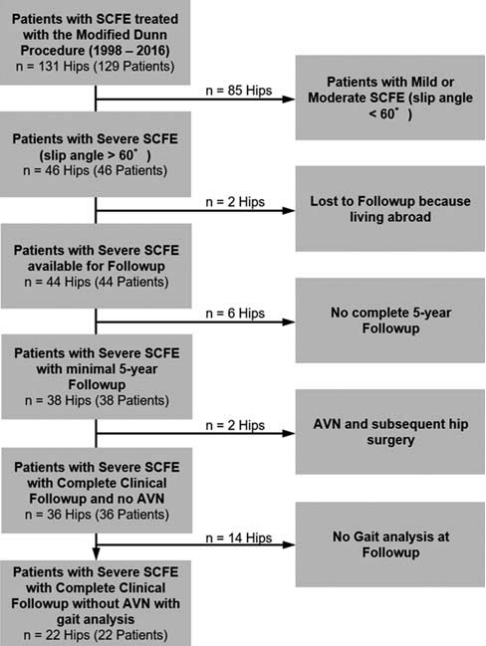
Flow chart of the patient series is shown. SCFE indicates slipped capital femoral epiphyses.

An IRB-approved retrospective analysis of 22 patients (22 hips) was performed. All patients were treated with a modified Dunn procedure for severe SCFE (slip angle ≥60 degrees according to Southwick^1^) between 1999 and 2016. During this time period, 131 hips were treated with a modified Dunn procedure for anatomic alignment in our institution (Fig. 1). All hips with severe (46 hips) and moderate displacement (slip angle between 30 and 60 degrees) were treated with the modified Dunn procedure. During this time period, all patients with severe SCFE that were treated with a modified Dunn procedure underwent prophylactic pinning of the contralateral side. The mean age was 13±1 years (range: 11 to 15 y, Table 1). Seven of 22 hips (32%) presented with unstable slips. Mean preoperative slip angle was 64±8 degrees (range: 60 to 90, Table 1).

**TABLE 1:** Demographic Information of the Patients With Severe SCFE and of the Volunteers is Shown

Parameter	SCFE Patients	Volunteers	*P*
Total hips (patients)	22 (22)	36 (18)	
Age at follow up (y)	22±3 (17-30)	29±6 (18-39)	NS
Age at operation (y)	13±1 (11-15)	NA	
Sex (% male of all hips)	68	55	NS
Side (% left of all hips)	86	50	
Height (cm)	160±8 (149-175)*	176±11 (154-191)	NS
Weight (kg)	65±14 (39-88)*	75±18 (44-120)	NS
Body mass index (kg/m^2^)	26±5 (20-34)*	24±4 (19-35)	NS
Follow up time (y)	9±2 (6-15)	NA	
Preoperative slip angle (degrees)	64±8 (60-90)*	NA	
Unstable hips according to Loder classification (% unstable of all hips)	7 (32)	NA	
Severe SCFE based on slip angle >60 degrees (% of all hips)	100	NA	
Classification based on the duration of symptoms (% of all hips)			
Acute	4 hips (18%)	NA	
Acute on chronic	12 hips (55%)	NA	
Chronic	6 hips (27%)	NA	

Continuous values are displayed as mean±SD (range).

* Values at time of operation.

NS indicates not significant; SCFE, slipped capital femoral epiphyses.

Gait analysis was performed at follow up of 22 patients (22 hips) treated with a unilateral modified Dunn procedure for severe SCFE (between 2002 and 2011). Of 46 patients with severe SCFE (Fig. 1), 2 patients were lost to follow up because they live abroad, 6 patients had no complete 5-year follow up and 2 patients had avascular necrosis of the femoral head. The remaining 36 patients were invited for routine clinical and radiographic follow up examination. Of them, 14 hips refused gait analysis (Fig. 1). This resulted in 22 hips with gait analysis at follow up (mean 9±2 y, range: 6 to 15, Table 1). These 22 patients were included in a previous study.^11^ The gait analysis and clinical outcome was evaluated and was compared with the contralateral side. All patients exhibited a heel-toe gait and did not complain of pain during level walking. There were no other existing lower extremity conditions (besides the painful hip) that affected the ability to walk comfortably and independently. None of the patients sustained from an underlying neurological disorder that altered their gait pattern. As control group, 36 feet of 18 healthy asymptomatic volunteers with a mean age of 29±6 (18 to 39) years were used. Preoperative analysis of FPA was not possible because some patients could not walk without crutches or could not weight-bearing on the affected side (32% had unstable SCFE) or because of the short-time between diagnosis and surgery (acute SCFE).

Gait analysis was performed using an instrumented walkway system (GAITRite; CIR Systems Inc., Franklin, NJ) to measure the FPA. The GAITRite system is a computer-based instrumented roll-up walkway with embedded pressure sensors that has been developed to measure spatial and temporal gait characteristics.^12^ The roll-up walkway with 18,432 embedded pressure sensors^12^ used for this study is 6 m long. The walkway’s active measurement area is 61 cm wide and 488 cm long. Sensors are arranged in a grid pattern (48×384) and placed 1.27 cm on center. The sampling rate of the system used varies between 32.2 and 38.4 Hz. Data are uploaded to a computer, and automatic footstep identification and calculation of parameters are made. This system provides quantitative information about the patient’s gait. Several authors reported the validity of the GAITRite system for measuring both spatial and temporal characteristics.^12^

The main outcome parameter was the FPA. This is a common parameter measured during gait analysis to detect in-toeing and out-toeing gait. The FPA was defined as the angle of out-toeing of the foot during stance phase compared with the line of gait progression.^6^ Normal FPA was defined −5 to 15 degrees. Out-toeing was defined as a FPA >15 degrees. In-toeing was defined as FPA<−5 degrees. The normal FPA in children is an out-toeing angle of the foot that ranges from 5^13^ up to 15 degrees,^14^ and others reported a normal FPA of 8 degrees.^15^ In-toeing was defined <−7 degrees and out-toeing was defined >20 degrees by others.^15^ Postoperative slip angle was compared with FPA at follow up.

The operative technique of the modified Dunn procedure was described in previous publications.^5^,^16^ In short, the surgical dislocation of the hip with an osteotomy of the greater trochanter was performed. An extended retinacular soft-tissue flap^17^ was developed for preservation of the blood supply to the femoral head. The capital epiphysis was first completely separated from the femoral neck, which allows full exposure of the femoral neck and visualization of the posteroinferior callus formation on the neck.^11^ This callus formation was removed completely to avoid tension on the terminal branches of the deep branch of the medial femoral circumflex artery. The femoral head was manually stabilized and the remaining epiphysis of the femoral head was removed.^18^ After gentle reduction of the femoral head back onto the femoral neck, the femoral head was stabilized using a threaded wire inserted in a anterograde manner through the fovea capitis.^18^ In addition, the femoral head was stabilized with a second threaded wire placed in a distal to proximal direction under fluoroscopic control.^18^ Epiphyseal perfusion was checked using a 2 mm drill hole to observe bleeding. Aftercare included 6 to 8 weeks partial weight-bearing for healing of the trochanteric osteotomy.^11^

Self-reported outcome instruments were collected with questionnaires using questions regarding the affected hip for the Harris hip scores (HHS) and the Hip Disability and Osteoarthritis Outcome Score (HOOS). For the HHS, the maximum is 100 points; a score of 91 to 100 points corresponded to excellent hip function; 81 to 90 points, good function; 71 to 80 points, fair function; and ≤70 points, poor hip function. The HOOS consists of 40 items assessing five dimensions: pain, function in activities of daily living, function in sport and recreation, hip-related quality of life and other symptoms. Each subscale has a score from 0 to 100, where 100 indicates no problem and 0 indicates extreme problem. The Merle d’Aubigné and Postel score has a maximum of 18 points.

Functional assessment of the hips included postoperative hip range of motion in flexion, abduction, adduction and internal (IR) and external rotation (ER) assessed with the hip in 90 degrees of flexion. These information were recorded from the medical records. Postoperative ROM was compared with FPA. Different observers performed the clinical evaluations at follow up. However, substantial interobserver and intraobserver agreement has been reported for ROM and the Drehmann’s sign in patients with SCFE.^19^ Clinical and radiographic information at follow up were evaluated by one of the authors (blinded) not involved in the clinical care of the patients. The anterior impingement test (also called FADIR test) was considered positive, if inguinal hip pain could be reproduced in forced flexion and internal rotation. Preoperative ROM is very difficult to measure, especially for severe and unstable SCFE. Preoperative ROM was not available for all patients and was not included.

A sample size calculation and power-analysis was performed for continuous variables of 2 groups with a level of significance of 5% and beta error of 10%, given previously reported mean values for FPA of 13 degrees for volunteers^20^ and 25 degrees for patients with SCFE.^10^ This resulted in 10 patients per group (clincalc.com, accessed on September 7, 2021).

### Statistical Analysis

Statistical analysis was performed with software Winstat (R. Fitch Software, Bad Krozingen, Germany). Normal distribution was tested using the Kolmogorov-Smirnov test. Because the data were normally distributed, analysis of variance was used for continuous data (eg, FPA). To compare demographic and radiographic data among the 3 groups, a Kruskal-Wallis test was used; if significant, we used the Mann-Whitney *U* test to compare each of the combinations of 2 groups. To compare binominal demographic data and the prevalence of out-toeing among the 3 groups, we used a χ^2^ test was used; if significant, the Fisher exact test was used to compare among each of the combinations of two groups.

## RESULTS

**FIGURE 2:**
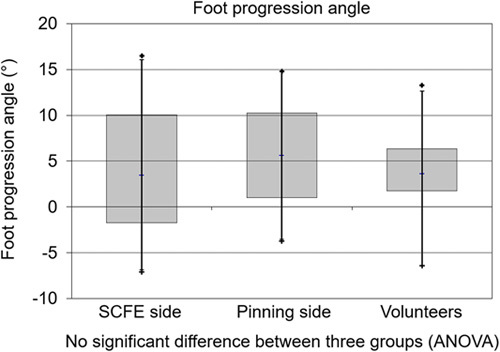
Boxplots of the foot progression angle (FPA) of the 3 groups are shown, no significant difference between the 3 groups were found. ANOVA indicates analysis of variance; SCFE, slipped capital femoral epiphyses.

**FIGURE 3:**
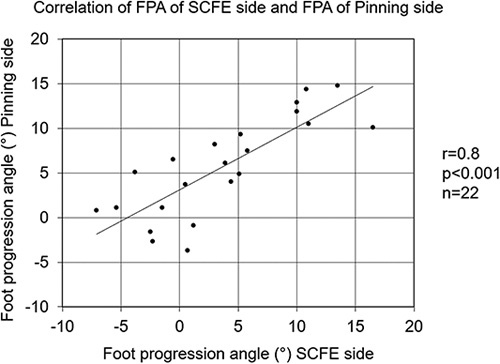
Correlation of the foot progression angle (FPA) of the slipped capital femoral epiphyses (SCFE) side and the FPA and of the pinning side is shown. A significant (*P*<0.001) and good (*r*=0.8) correlation was found indicating symmetrical FPA.

1. Mean FPA of the SCFE side was 3.6±6.4 degrees (range: −7 to 17) and was not significantly different compared with 5.6±5.5 degrees (range: −4 to 15) of the contralateral side and compared with mean FPA of the control group (4.0±4.5 degrees (range: −6 to 13), Figure 2). A significant (*P*<0.001) and good (*r*=0.8) correlation was found between the FPA of the SCFE patients and the FPA of the contralateral side (Fig. 3). The mean side-to-side difference of the FPA (2.0 degrees) for SCFE patients compared with the controls (2.2 degrees) was not significantly different.
2. Of the 22 SCFE hips, 19 hips (86%) had normal FPA, 2 hips had in-toeing (9%) and 1 hip had out-toeing (5%). The prevalence of out-toeing of the foot was not significantly different between patients with severe SCFE and the 2 other groups. Two volunteers had in-toeing (6%) and none of them had out-toeing. Of the contralateral side, no in-toeing and no out-toeing was present.
3. Mean HHS was 93±11 points (66 to 100), mean HOOS was 91±10 points (67 to 100), mean Merle d’Aubigné and Postel score was 18 points (range: 16 to 18, Table 2) and WOMAC was 4±8 at follow up. Three patients (14%) had HHS <80 points and walked with normal FPA. The 2 patients with in-toeing and 1 patient with out-toeing had HHS >95 points.

**TABLE 2:** Patient Reported Outcome Scores at Most-recent Follow-up of Patients That Underwent the Modified Dunn Procedure for Severe SCFE are Shown

Patient Reported Outcomes	Value
Modified Harris hip score	93±11 (66-100)
HOOS total score	91±10 (67-100)
Pain	95±8 (68-100)
Function and daily living	97±7 (72-100)
Sports and recreational activities	90±17 (38-100)
Symptoms and stiffness	90±13 (50-100)
Quality of life	84±15 (50-100)
UCLA activity score	8±1 (5-10)
Normalized WOMAC score	4±8 (0-28)
Merle d’Aubigné and Postel score	18±1 (16-18)
Hips with HHS <80 points (% of all hips)	3 (14)

Continuous values are expressed as mean and range in parenthesis.

HOOS indicates Hip Disability and Osteoarthritis Outcome Scores; SCFE, slipped capital femoral epiphyses; UCLA, University of California; WOMAC, Western Ontario and McMaster Universities Osteoarthritis Index.

Mean flexion was 107±10 degrees and IR in 90 degrees of flexion was 37±17 degrees at follow up (Table 3). We found a significant correlation between FPA and external rotation in 90 degrees of flexion (*P*=0.042, *r*=0.39) of the SCFE patients at follow up. No significant correlation between postoperative flexion or internal rotation in 90 degrees of flexion and the FPA was found. No significant correlation between postoperative slip angle or alpha angle and the FPA was found. Mean postoperative slip angle was 8±4 degrees (Table 4).

**TABLE 3:** Range of Hip Motion at Most-recent Follow-up is Shown for SCFE Patients

Range of Hip Motion at Follow up	Value
Flexion (degrees)	107±10 (90-120)
Internal rotation in 90 degrees of flexion (degrees)	37±17 (10-70)
External rotation in 90 degrees of flexion (degrees)	59±16 (40-80)
Abduction in extension (degrees)	35±8 (25-50)
Adduction in extension (degrees)	22±7 (15-30)
Positive Drehmann’s sign (% of all hips)	0

Continuous values are expressed as mean and range in parenthesis.

SCFE indicates slipped capital femoral epiphyses.

**TABLE 4:** Radiographic Results at Follow up of 22 SCFE Patients are Shown

Parameter	SCFE Patients
Slip angle at follow up (degrees)	8±4 (1-16)
Alpha angle on lateral view at follow up (degrees)	39±13 (26-71)
Alpha angle on AP view at follow up (degrees)	55±19 (31-94)
Articulotrochanteric distance (mm)	42±8 (25-57)
Minimum joint space width (mm)	3±0 (3-4)

Continuous values are expressed as mean and range in parenthesis.

AP indicates anteroposterior; SCFE, slipped capital femoral epiphyses.

## DISCUSSION

The aim of this study was to investigate gait analysis and the prevalence of out-toeing of patients with severe SCFE after treatment with the modified Dunn procedure. Therefore, the primary purpose of this study is to determine if severe SCFE patients walk normal. A secondary purpose was to investigate if the prevalence of out-toeing was different in these patients compared with contralateral side and compared with a control group. Last, hip outcome scores were evaluated to determine clinical outcome.

Most importantly, we found that the mean FPA of patients with severe SCFE treated with a modified Dunn procedure showed no difference compared with control group and compared with contralateral side (Fig. 2). The mean side-to-side difference of the FPA (2.0 degrees) for SCFE patients was similar compared with control group. The prevalence of out-toeing of the foot was not significantly different between patients with severe SCFE and the 2 other groups.

This study investigated gait analysis in patients with severe SCFE treated with a modified Dunn procedure. Few studies investigated gait outcome of patients with severe SCFE. For other pediatric diseases, previous investigations have been performed to study gait and FPA and conditions affecting gait.^8^ In a recent systematic review investigating patients with stable SCFE,^21^ no information on gait analysis was provided. A previous study investigated gait outcomes using 3D gait analysis at 1-year follow up in patients with severe SCFE treated with flexion-rotation osteotomy and described an improved gait deviation index^10^ and decreased FPA (from 26.5 degrees preoperatively to 10 degrees postoperatively). Another study reported a FPA of 3 to 4 degrees of patients with severe SCFE.^22^ Others reported improved gait after flexion-valgus intertrochanteric osteotomy in a group of 11 patients with severe SCFE at short-term follow up (16 mo). A previous study investigated preoperative gait analysis of SCFE patients and evaluated the relationship between slip severity, function, and gait disturbances.^23^ They found as slip severity increased, there was greater pelvic obliquity (down on affected side), increased hip ER, increased external FPA, and decreased knee flexion.^23^ Another study found an abnormal gait profile for moderate to severe SCFE patients after in situ pinning.^24^ More recently, gait analysis of mild and moderate SCFE patients treated with in situ pinning showed an increase in hip extension moment.^25^

Comparing the mean FPA of the SCFE side to contralateral side and to control group, no significant difference (Fig. 2) was found. Compared with another study^26^ investigating healthy asymptomatic volunteers, we found a comparable FPA. They described a normal FPA of 4.5±5.6 degrees for males and 1.4±5.4 degrees for females.^26^ Another study^20^ described a higher mean FPA for asymptomatic volunteers (13 to 14 degrees), but without instrumented gait analysis. Comparing the BMI of the patients and volunteers, we found no significant difference. This could be different in other countries (eg, the USA), where overweight SCFE patients were described.

This study has limitations. First, the FPA was captured during the stance phase only. However, the stance phase is more robust for measurements of the FPA compared with the toe off phase, for example.^27^ Second, we did not quantify any potential concomitant foot deformity. However, based on the clinical examination, none of our patients presented with foot pain, which should therefore not jeopardize our results. Third, our measurements were done at 1 single time point. Theoretically, the FPA could change during daytime and with activities of daily living. Given the reported mean error of <1 degrees for the measurement of FPA at 2 different time points^12^ and the high accuracy of the Gaitrite system,^12^ this should not influence our data to a relevant degree. No information on femoral version and tibial torsion^6^,^28^ was available for the evaluated SCFE patients and no information on alpha angle was available after contralateral prophylactic pinning.^29^ The clinical evaluation was done by different observers because of the retrospective design and a follow up period of almost 10 years (2002 to 2011). This could include a potential bias. However, substantial interobserver and intraobserver agreement has been reported for the Drehmann sign in patients with SCFE.^19^ Therefore, we believe this should not have biased our clinical results to a relevant degree. All hips with severe SCFE during the time period were uniformly treated with the modified Dunn procedure, independent whether they were stable or unstable. In other studies, only SCFE patients with severe and stable SCFE deformity were included.^30^

## CONCLUSIONS

Gait analysis of patients that underwent the modified Dunn procedure for severe SCFE showed mostly normal FPA. The prevalence of out-toeing gait was not significantly increased compared with control group. High hip scores were found at long term follow up. Although one SCFE patient had out-toeing gait at follow up, good hip scores were noted. These findings are in contrast to previous studies and could be important for surgeons treating SCFE patients.
